# Effects of Maren Pills on the Intestinal Microflora and Short-Chain Fatty Acid Profile in Drug-Induced Slow Transit Constipation Model Rats

**DOI:** 10.3389/fphar.2022.804723

**Published:** 2022-04-12

**Authors:** Yu Zhan, Yong Wen, Li-juan Du, Xiao-xiang Wang, Shi-yu Tang, Peng-fei Kong, Wei-guo Huang, Xue-gui Tang

**Affiliations:** ^1^ Department of Anorectal, Affiliated Hospital of Integrative Chinese Medicine and Western Medicine of Chengdu University of TCM, Chengdu, China; ^2^ Department of Anorectal, Chengdu First People’s Hospital, Chengdu, China; ^3^ Department of Anorectal, Hospital of Chengdu University of Traditional Chinese Medicine, Chengdu, China; ^4^ Department of Traditional Chinese Medicine, The Affiliated Hospital of Southwest Medical University, Luzhou, China; ^5^ Department of Anorectal, The Third People’s Hospital of Chengdu, Chengdu, China; ^6^ Department of Digestive medicine, Chengdu First People’s Hospital, Chengdu, China; ^7^ Department of Gastrointestinal Surgery, Affiliated Hospital of North Sichuan Medical College, Nanchong, China; ^8^ Department of Anorectal Affiliated Hospital of North Sichuan Medical College, Nanchong, China

**Keywords:** slow transit constipation, Maren pills, gut microbiota, intestinal metabolism, 5-HT pathway

## Abstract

**Background:** Slow transit constipation (STC) is becoming a common and frequently occurring disease in today’s society, and it is necessary to explore the safe and effective treatment of STC.

**Method:** Our study aimed to investigate whether the laxative effect of Maren pills (MRW) is associated with the regulation of intestinal microflora and intestinal metabolism in the colon. Loperamide hydrochloride-induced STC rats received MRW intragastrically for two consecutive weeks to evaluate the laxative effect of MRW involving the regulation of intestinal microflora, intestinal metabolism, and 5-HT signaling pathway. Intestinal microflora was detected by 16s rDNA sequencing, intestinal metabolism of short-chain fatty acids (SCFAs) was detected by HPLC, and the 5-HT signaling pathway was detected by WB, ELISA, immunofluorescence, and immunohistochemical analysis.

**Results:** Our results revealed that the treatments with MRW increased not only the body weight, 24-h fecal number, 24-h wet fecal weight, 24-h dry fecal weight, fecal water content, and the intestinal propulsion rate but also the colonic goblet cell number, colonic Muc-2 protein expression, and colonic mucus layer thickness in the STC model rats. Moreover, MRW activated the 5-HT pathway by increasing the levels of 5-HT, 5-HIAA, 5-HT4R, CFTR, cAMP, and PKA in the colon tissue of STC rats. The 16S rDNA sequencing results showed that MRW improved the colonic microflora structure in colonic contents of STC rats, mainly by increasing *Lactobacillus* and decreasing *Prevotella*. Finally, we found that MRW regulated the SCFA metabolism in the colonic contents of the STC rats, mainly by increasing the contents of acetic acid, propionic acid, and butyric acid; the relative abundance of *Lactobacillus* was positively correlated with either contents of acetic acid, propionic acid, and butyric acid, and the relative abundance of *Clostridium* was negatively correlated.

**Conclusion:** Our study further showed that MRW could improve constipation in STC rats, and the mechanism may be by regulating the intestinal microflora structure and improving the metabolism of SCFAs.

## Introduction

Slow transit constipation (STC) is a common type of functional constipation, characterized by infrequent, difficult defecation and hard dry stool (Hanson et al., 2019). Although STC is not life-threatening, it can adversely impair patient’s life quality and pose a high economic burden on the society (Inoue et al., 2017). In recent years, the STC has been reported to be linked with aberrant gastrointestinal tract, which is usually caused by dysfunction of interstitial cells of Cajal (ICC) (Zhu et al., 2016) and an abnormal 5-hydroxytryptamin (5-HT) signal (Guarino et al., 2011; Wu et al., 2020). In addition, increasing evidence suggests that intestinal microflora is another vital factor that is related to the development of STC (Ding et al., 2018; He et al., 2020).

The intestinal microflora has been believed to be related to the pathogenesis of many diseases, such as inflammatory bowel disease (Vich Vila et al., 2018), virus infection (Groves et al., 2020), metabolic disease (Zhong et al., 2016), and constipation (Zhuang et al., 2019). A previous study demonstrated decreased relative abundance of *Bifidobacterium* and *Lactobacillus* along with increased abundance of Desulfovibrionaceae in patients with constipation (Zhuang et al., 2019). In addition, a remarkable reduction of butyrate-producing bacteria, such as *Faecalibacterium*, Ruminococcaceae, and *Roseburia*, was also observed in constipation, which was further confirmed by reduced production of short-chain fatty acids (SCFAs), especially butyric acid, caused by constipation (Mancabelli et al., 2017; Zhuang et al., 2019; He et al., 2020). Intestinal metabolites may bridge the intestinal microflora to STC. It has been reported that the altered intestinal microflora was associated with constipation-related bile acids (Bas), SCFAs, tryptophan metabolism, as well as intestinal integrity and motility (Zhang et al., 2021). As the main metabolite of intestinal microflora, SCFAs have been demonstrated to promote 5-hydroxytryptamine (5-HT) biosynthesis from colonic enterochromaffin cells (ECs), thus modulating gastrointestinal motility (Yano et al., 2015). Some gut bacteria could metabolize tryptophan into tryptamine, in which tryptamine could activate the cAMP-dependent chloride channel in colonic epithelium, leading to increased intestinal secretion and faster intestinal transit (Bhattarai et al., 2018). These discoveries suggest that the regulation of intestinal microflora and intestinal metabolites may be promising therapies for STC.

Maren pills (MRW) are compound medicine composed of six Chinese herbs, including Semen cannabis, Rhubarb, Apricot kernel, *Magnolia officinalis*, *Paeonia lactiflora* Pall, and Fructus aurantii immaturus (Table 1) (Zhan et al., 2020). As a widely used herb clinically, Maren pills have been demonstrated to improve symptoms of constipation patients and increase defecating frequency (Yin, 2016). Moreover, our previous study also confirmed the therapeutic role of MRW in STC rats *via* regulating AQP3 and CFTR signaling pathways (Zhan et al., 2020). However, the influence of MRW on the intestinal microflora and intestinal metabolite in the context of STC remains unknown.

**TABLE 1:** Information of MRW plant contents.

Herbs name	Plant names	Family	Medicinal part
Semen cannabis	*Cannabis sativa L*	Cannabaceae *Martinov*	Seeds
Rhubarb	*Rheum palmatum L*	Polygonaceae *Juss*	Rhizome
Apricot kernel	*Prunus armeniaca L.var.ansu Maxim.*, Prunus sibirica L., Prunus mandshurica (Maxim.) Koehne, and Prunus armeniaca L	Rosaceae *Juss*	Seeds
Magnolia officinalis	*Magnolia officinalis Rehder and E.H.Wilson*	Magnoliaceae *Juss*	Bark and root bark
*Paeonia lactiflora* Pall	*Cynanchum otophyllum C.K.Schneid*	Apocynaceae *Juss*	Root
Fructus aurantii immaturus	*Citrus aurantium L*	Rutaceae *Juss*	Fruit

Therefore, in this study, we investigated the anti-STC effects of MRW using a loperamide hydrochloride-induced STC model, followed by a gut microbial analysis and intestinal metabolite quantification to identify the molecular mechanism of Maren pills in the treatment of STC.

## Materials and Methods

### Animals and Experimental Design

A total of 72 healthy male SD rats (210–230 g, 8 weeks old) were purchased from the Animal Center of West China Medical College, Sichuan University (Chengdu, China). All rats were housed in cages with free access to food and water at controlled temperature (23 ± 2°C), humidity of 50–55%, and 12-h light–dark cycle. Loperamide hydrochloride was bought from Xian Janssen Co., Ltd. (lot: 171205792, Xian, China). MRW were obtained from Chongqing Taiji Industry Co., Ltd. (lot: Z50020517, Chongqing, China). Chemical compositions of MRW were confirmed using ultra-performance liquid chromatography–mass spectrometry (UPLC-MS) (the detection method is shown in the Supplementary Material), and the UPLC-MS results are shown in Supplementary Table S1. Among the chemical compositions of MRW, the content of sulfuric acid monoesters was the highest.

After 1 week of adaptive feeding with a basal diet, the rats were randomly divided into six groups with 12 rats in each group as follows: control group, model group, low dose of MRW group (L-MRW), medium dose of MRW group (M-MRW), high dose of MRW group (H-MRW), and mosapride group (positive). The rats in the model, MRW-treated, and positive groups received 1.5 mg/kg/d loperamide hydrochloride by intragastric administration in the morning, and the treatment was given for 2 weeks to establish the STC model (Lee et al., 2012; Narita et al., 2020; Li et al., 2021; Liu and Zhi, 2021). For the MRW-treated groups, rats were gavaged daily afternoon with MRW during the 2-week period using 0.5, 1, and 2 g/kg body mass dose in their corresponding group, respectively. For the positive group, rats were gavaged daily afternoon with 1.6 mg/kg of mosapride during the 2-week period. The control group was administered with an equal volume of saline by gavage. The body weight was recorded every 2 days. The 24-h feces were collected after the last treatment for further analysis. Rats were anesthetized and then killed by cervical dislocation to harvest colon tissue and contents for further analysis.

### 24-h Defecation and Fecal Water Content

At the end of the last treatment, rats were housed individually in metallic cages to collect feces once an hour for 24 h, and the feces number and weight were recorded. The fecal water content was calculated after drying the feces in a desiccator at 60°C for 12 h, according to the equation: (wet weight−dry weight)/wet weight ×100%.

### Measurement of Intestinal Transit Rate

To evaluate the intestinal motility, the rats (n = 6) in each group were gavaged with a charcoal meal (20 ml/kg, 3% suspension of activated charcoal in 0.5% aqueous methylcellulose) after 30 min at the end of the last treatment. After 40 min, rats were euthanized. The intestinal transit rate was calculated as follows: traveled distance of activated charcoal in the intestine (cm)/full length of the small intestine (cm) ×100%.

### Histological Analysis

Colon tissues were initially fixed in 4% paraformaldehyde, embedded into paraffin, and sectioned into 5-μm slides. After deparaffinization and rehydration, hematoxylin and eosin (HE) staining was performed to measure histological morphology. Periodic acid–Schiff (PAS) staining was used to measure the colonic mucus thickness.

### Immunohistochemical Staining

Immunohistochemical staining was conducted to analyze the expression of 5-HT using a primary antibody against the protein. First, the paraffin sections of colon tissues were incubated with 3% hydrogen peroxide and blocked with a regular blocking solution (5% skim milk powder). Then the sections were incubated with the primary antibody (anti-5-HT, 1:500; lot: FNab09928, FineTes, Wuhan, China) at 4°C for 12 h. After washing three times, all sections were incubated with secondary antibodies at 37 °C for 2 h. Finally, DAB was used for chromogenic detection, followed by washing the sections. The immunohistochemical results were observed.

### Immunofluorescence Staining

Immunofluorescence staining was used to assess the expression and distribution of 5-HT4R and CFTR in the colonic epithelium. The paraffin sections of the colon tissues were blocked with 2% BSA for 30 min. Primary antibodies against 5-HT_4_R (1:500, ab60359, Abcam, United States) and CFTR (1:500, ab131553, Abcam, United States) were added and incubated overnight. After three washes, the anti-Cy3 IgG secondary antibody or the anti-FITC IgG secondary antibody was added and incubated for 2 h. Finally, DAPI nuclei staining was performed. Images were captured using a fluorescent microscope (Olympus BX51; Olympus Corp., Tokyo, Japan) at magnification ×200 and ×400.

### ELISA

The production of 5-HT and TPH-1 in the colon tissue was measured using mouse 5-HT ELISA (lot: ER1463, FineTes, Wuhan, China) and TPH-1 ELISA kits (lot: ER1409, FineTes, Wuhan, China), following the manufacturer’s instruction.

### Western Bolt Analysis

Total proteins were extracted from the colon tissues using a RIPA lysis buffer (Beyotime, Beijing, China), and a BCA protein assay kit (Vazyme, Nanjing, China) was used to measure protein concentration. After separating by 10% SDS-PAGE and transferring on polyvinylidene fluoride (PVDF) membranes, the samples were blocked by 5% skim milk and kept for 2 h at room temperature. Then the samples were incubated with primary antibodies for overnight at 4°C. The information of the antibodies is as follows: Muc-2 (1:1000, ab272692, Abcam, United States), 5-HT_4_R (1:500, ab60359, Abcam, United States), CFTR (1:500, ab181782, Abcam, United States), cAMP (1:5000, ab76238, Abcam, United States), and PKA (1:1000, ab75991, Abcam, United States). Next, the samples were subsequently incubated with secondary antibodies for 2 h at room temperature after washing three times. Finally, a chemiluminescence detection system was used to detect the samples, and ImageJ was used to quantify the intensity of the bands. β-actin was used as an internal reference.

### 16S rDNA Sequencing and Analysis

Microbial DNA from colonic content samples was extracted by using the ZR Fecal DNA Extraction Kit (Zymo Research, CA, United States), and the V3-V4 region was amplified using the universal primers (319F: 5′-ACT​CCT​ACG​GGA​GGC​AGC​AG-3′; 806R: 3′-ACT​CCT​ACG​GGA​GGC​AGC​AG-5′). The samples were sent to the Shanghai Personal Biotechnology Co., Ltd. (Shanghai, China) for pooling and paired-end sequencing on an Illumina MiSeq sequencer (Illumina). The Quantitative Insights Into Microbial Ecology (QIIME, v1.8.0) pipeline and the Quantitative Insights into Microbial Ecology (QIIME) software packages, version 1.9.1 were used to process the sequencing data and microbial composition analysis. By statistical analysis of the ASV/OTU table after extraction, the specific composition table of microbial community in each sample at each classification level can be obtained. A principal components analysis (PCA) was performed using the following website: https://www.omicshare.com/tools/index.php/Home/Soft/pca, and the heat map of genera level community composition was analyzed by using the following website: https://www.bioincloud.tech/standalone-task-ui/cor_heatmap. Metabolic pathway statistics were analyzed by the KEGG Pathway database (http://www.genome.jp/kegg/pathway.html) and the MetaCyc database. The original sequencing data were uploaded to SRA database using the following link: https://www.ncbi.nlm.nih.gov/sra/PRJNA792032.

### Determination of Intestinal Metabolites in Colonic Contents

Finally, SCFAs in colonic contents were detected, according to a previous study (Fan et al., 2021). In brief, the samples were extracted by adding 1,000 μL of ethanol (containing 0.5% hydrochloric acid, v/v) and 10 μL of internal reference, then vortexing and ultrasonicating for 40 min, and finally centrifuging at 14,000 rpm for 10 min. Next, the extracted samples were detected by Agilent 7890-5977 gas chromatography–mass spectrometry (Agilent Technologies, Santa Clara, CA, United States). The chromatographic column was a DB-FFAP (30 m × 0.25 mm i.d., 0.25-μm film, Agilent Technologies). The parameter settings were the same to the previous study (Fan et al., 2021). Standard reference database 1A was used to match the results. The levels of acetic acid, propionic acid, isobutyric acid, butyric acid, isovaleric acid, valeric acid, and caproic acid were detected.

### Statistical Analysis

All data are shown as mean ± SD. GraphPad Prism 7 (GraphPad Software Inc., United States) and SPSS 19.0 (IBM SPSS software, United States) were used to analyze data, and the statistical differences among the five groups were determined by one-way ANOVA. If the data fitted the homogeneity of variance, LSD analysis was selected; if not, Tamhane’s T2 analysis was selected. Repeated-measures ANOVA was used to analyze rats’ body weight by SPSS 19.0. Linear, quadratic, and cubic models were used to analyze the 24-h fecal number, 24-h wet fecal weight, 24-h dry fecal weight, fecal water content, and intestinal propulsion by SPSS 19.0. Pearson’s correlation test was analyzed by SPSS 19.0. *p* < 0.05 was considered statistically significant.

## Results

### MRW Improved Constipation of the STC Rats

As shown in Table 2, since the fourth day, the body weight of the STC model rats was significantly lower than that of control rats. Since the sixth day, the body weights of rats in the M-MRW, H-MRW, and positive groups were significantly higher than that of the STC model rats. On the 14th day, the body weights of rats in the L-MRW, M-MRW, H-MRW, and positive groups were significantly higher than that of the STC model rats. On the eighth, 12th, and 14th days, the body weight of rats in the L-MRW was significantly lower than that of the positive rats. There was no significant difference in the body weight between the H-MRW and positive rats in this experiment. Moreover, the analysis of Pillai’s Trace and Roy’s greatest root in the repeated-measures ANOVA showed that time*MRW dose was an important factor in the MRW treatment. As shown in Table 3, the 24-h fecal number, 24-h wet fecal weight, 24-h dry fecal weight, fecal water content, and intestinal propulsion of the STC model rats were obviously decreased, and those in the L-MRW, M-MRW, and H-MRW rats were increased compared to the STC model rats. Moreover, the 24-h fecal number, fecal water content, and intestinal propulsion of the H-MRW group were similar to those in the positive group. Otherwise, the data in the 24-h fecal number, 24-h wet fecal weight, 24-h dry fecal weight, fecal water content, and intestinal propulsion presented results from a linear regression model.

**TABLE 2:** Changes in the body weight of the rats.

Time group	0d	2d	4d	6d	8d	10d	12d	14d
Control	217.18 ± 2.45	220.44 ± 7.06	224.30 ± 3.75^***^	224.97 ± 3.55^***^	226.01 ± 6.73^***^	228.83 ± 5.16^***^	232.50 ± 4.79^***^	233.46 ± 4.54^***^
Model	216.76 ± 3.43	214.55 ± 4.73	210.95 ± 3.78	209.87 ± 5.29	208.78 ± 6.82	205.37 ± 3.27	203.65 ± 8.53	200.75 ± 4.07
L-MRW	216.71 ± 6.14	215.05 ± 5.08	213.01 ± 3.64	214.29 ± 4.20	212.29 ± 5.77^$^	211.68 ± 2.62^*$$^	215.29 ± 3.48^*^	214.99 ± 4.21^***$$^
M-MRW	216.55 ± 2.49	215.83 ± 6.21	215.81 ± 5.30	219.96 ± 3.70^***^	219.70 ± 7.35^*^	215.30 ± 4.08^**$^	217.83 ± 6.30^**^	223.20 ± 2.77^***^
H-MRW	216.54 ± 4.29	215.06 ± 4.88	214.36 ± 6.09	217.21 ± 5.35^**^	221.84 ± 8.69^**^	221.70 ± 6.39^***^	221.53 ± 8.98^***^	225.62 ± 5.74^***^
Positive	217.02 ± 4.27	214.36 ± 4.81	215.29 ± 3.11	218.94 ± 3.73^**^	223.39 ± 7.64^**^	220.84 ± 5.42^***^	221.67 ± 10.1^***^	225.19 ± 6.27^***^
F	0.024	1.029	6.533	8.338	5.192	18.821	9.727	34.372
P	1.000	0.419	0.000	0.000	0.002	0.000	0.000	0.000
Pillai’s trace	Time		F	1.619
			P	0.210
	MRW dose*time		F	1.954
			P	0.028
Roy’s greatest root	Time		F	1.619
			P	0.210
	MRW dose*time		F	14.150
			P	0.000

Note: The data were normally distributed and determined by one-way ANOVA, following LSD analysis. Repeated-measures ANOVA was used to analyze the rats’ body weights among model, L-MRW, M-MRW, and H-MRW, groups. ^*^ *p* < 0.05, ^**^ *p* < 0.01, ^***^ *p* < 0.001 vs. model group; ^$^ *p* < 0.05, ^$$^ *p* < 0.01, ^$$$^ *p* < 0.001 vs. positive group.

**TABLE 3:** Defecation of rats.

	Control	Model	L-MRW	M-MRW	H-MRW	Positive	F	P	Model *p* Value		
									Linear	Quadratic	Cubic
Fecal number (24 h)	38.7 ± 1.2^***^	15.7 ± 2.2	25.8 ± 1.7^***$$^	26.7 ± 12.9^***^	32.2 ± 2.6^***^	31.7 ± 4.0^***^	51.402	0.000	0.438	0.000	0.000
Fecal weight (24 h) (g)	7.846 ± 0.785^***^	3.780 ± 1.210	5.188 ± 0.878^*$$$^	5.744 ± 2.951^**$$^	7.023 ± 1.131^***$^	8.319 ± 1.071^***^	16.312	0.000	0.633	0.000	0.001
Fry fecal weight (24 h)	5.94 ± 0.633^***^	3.246 ± 1.028	4.276 ± 0.873^*$$$^	4.497 ± 2.044^**$$^	5.332 ± 0.613^***$^	6.417 ± 0.987^***^	9.720	0.000	0.751	0.000	0.004
Water content (%)	24.33 ± 1.71^***^	13.86 ± 2.59	17.92 ± 3.80^$^	18.85 ± 6.73^**^	23.52 ± 5.53^***^	22.95 ± 3.98^**^	5.811	0.001	0.321	0.001	0.005
Propelling rate (%)	68.783 ± 3.143^***^	35.661 ± 2.462	45.748 ± 4.526^**$$$^	54.724 ± 4.083^**^	56.502 ± 4.325^***^	56.81 ± 7.62^***^	35.051	0.000	0.305	0.000	0.000

Note: The data were normally distributed and analyzed by one-way ANOVA. Linear, quadratic, and cubic model analyses were used to predict the MRW dose model among model,L-MRW, M-MRW, and H-MRW groups. ^*^ *p* < 0.05, ^**^ *p* < 0.01, ^***^ *p* < 0.001 vs. model group; ^$^ *p* < 0.05, ^$$^ *p* < 0.01, ^$$$^ *p* < 0.001 vs. positive group.

### MRW Improved Colonic Mucus Barrier in the STC Rats

**FIGURE 1:**
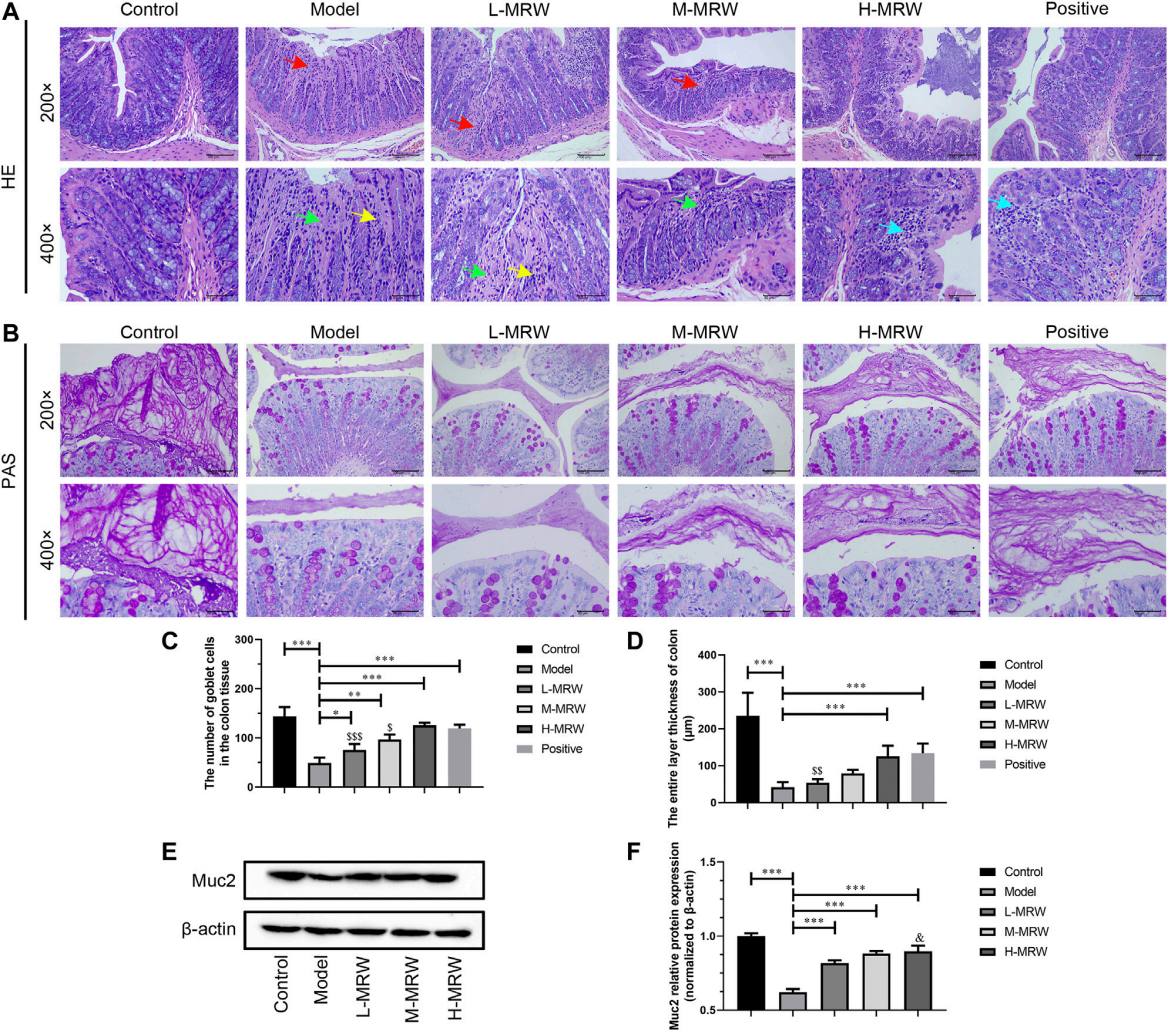
MRW improved the colonic mucus barrier in the STC rats. **(A)** HE staining results of colon tissue; red arrow: degeneration necrosis of mucosa; green arrow: adenomas necrosis; yellow arrow: goblet cell loss; and blue arrow: lymphocytic infiltration. **(B)** PAS staining results of colon tissue. **(C)** Number of goblet cells in colon tissue observed by HE staining. **(D)** Colonic mucus layer thickness observed by PAS staining. **(E)** WB results of Muc-2 protein expression in colon tissue. All data were normally distributed and analyzed by one-way ANOVA. ^*^ *p* < 0.05, **p < 0.01, ***p < 0.001 vs. model group; ^$^ *p* < 0.05, ^$$^ *p* < 0.01, ^$$$^ *p* < 0.001 vs. positive group; and ^&&^ *p* < 0.01 vs. L-MRW group.

As shown in Figure 1A,C, in the STC rats, the mucosal layer of the colon tissue was damaged, the epithelium cells were lost after its exfoliation, the intestinal gland in lamina propria was dissolved and necrotic, and the goblet cells in the intestinal gland were significantly reduced. Compared to the STC rats, the intestinal gland necrosis in the lamina propria of the colon tissue was improved and the goblet cells number in the intestinal gland was significantly increased in the L-MRW, M-MRW, H-MRW, and positive rats. Among these, H-MRW and positive groups showed similar obvious improvement effects on the structure of STC rats’ colon. The PAS staining results showed that L-MRW, M-MRW, H-MRW, and positive treatments all increased the colonic mucus layer thickness of the STC rats, and H-MRW and positive treatments had a higher increase in the colonic mucus layer thickness of STC rats’ colon (Figure 1B,D). As shown in Figure 1E, in the STC rats, the Muc-2 protein expression was decreased when compared with the control rats. After treatment with MRW, the Muc-2 protein expression in the L-MRW, M-MRW, and H-MRW rats were increased, compared with the STC rats.

### MRW Promoted the Secretion of 5-HT and Activated Its Receptor Pathway in STC rats

**FIGURE 2:**
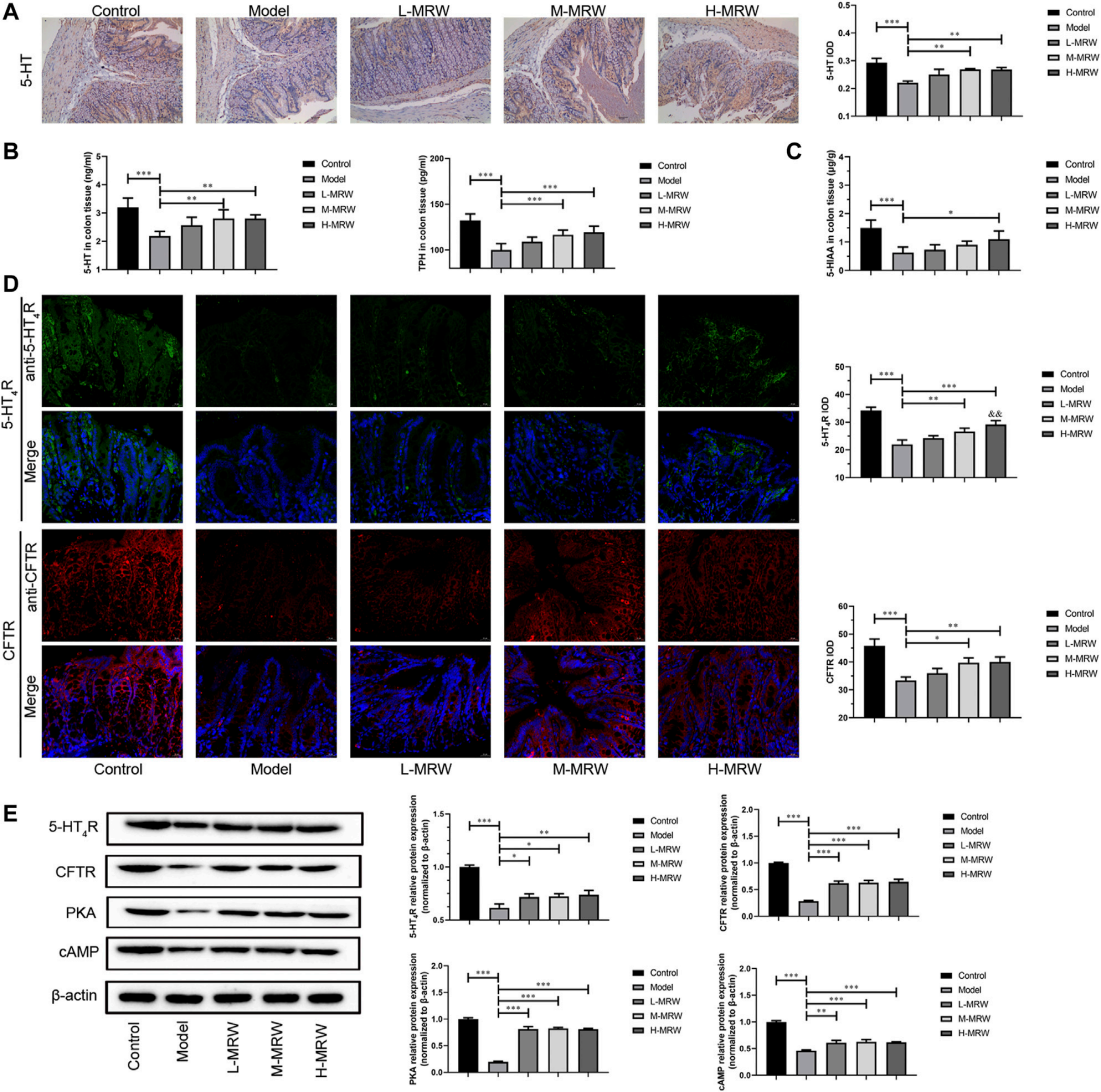
MRW promoted the secretion of 5-HT and activated its receptor pathway in the STC rats. **(A)** Immunohistochemical results of 5-HT in colon tissue. IOD: integral optical density. **(B)** ELISA results of 5-HT and TPH in colon tissue. **(C)** HPLC results of 5-HIAA in colon tissue. **(D)** Immunofluorescence results of 5-HT_4_R and CFTR in colon tissue. IOD: integral optical density. **(E)** WB results of 5-HT_4_R, CFTR, cAMP, and PKA in colon tissue. All data were normally distributed and analyzed by one-way ANOVA. ^*^ *p* < 0.05, ^**^ *p* < 0.01, ^***^ *p* < 0.001 vs. model group; ^&^ *p* < 0.05, ^&&^ *p* < 0.01, ^&&&^ *p* < 0.001 vs. L-MRW group.

As shown in Figure 2A, in the STC rats, the 5-HT expression was decreased when compared with the control rats. After the treatment with MRW, the 5-HT expression in the M-MRW and H-MRW rats were increased in comparison with the STC rats. As shown in Figure 2B, 5-HT and TPH-1 levels were decreased in the STC rats compared to the control rats, detected by ELISA, and those in the M-MRW and H-MRW rats were increased when compared with the STC rats. As shown in Figure 2C, the 5-HIAA level was decreased in the STC rats and increased in the H-MRW rats. As shown in Figure 2D, the expression of 5-HT_4_R and CFTR were decreased in the STC rats compared to the control rats and increased in the M-MRW and H-MRW rats compared to the STC rats. Otherwise, the expression of 5-HT_4_R in H-MRW rats was higher than that in L-MRW. As shown in Figure 2E, there were a decrease in the 5-HT_4_R, CFTR, cAMP, and PKA protein expression in the STC rats compared to the control rats, and an increase in those in the M-MRW and H-MRW rats compared to the STC rats was observed.

### Effects of MRW on Colonic Microflora in STC Rats

**FIGURE 3:**
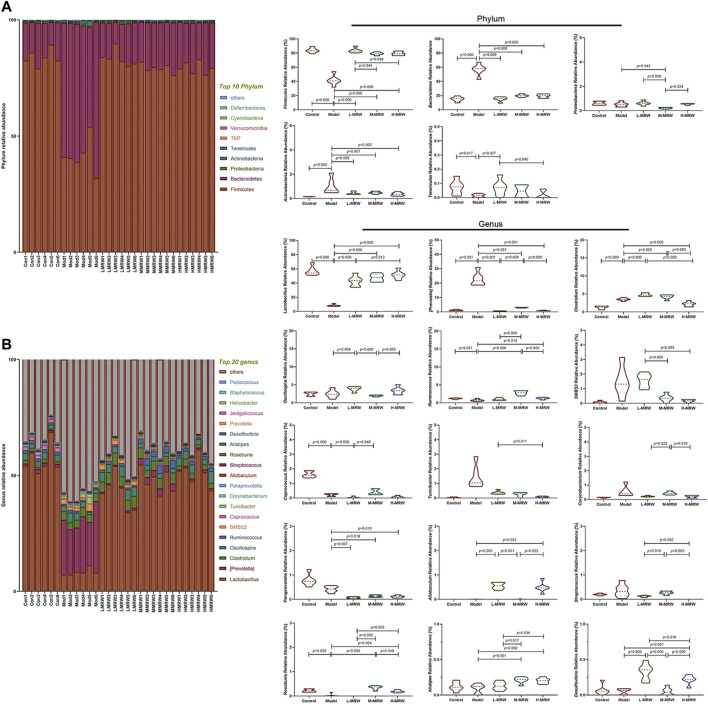
Microflora of the colon. **(A)** Microbial composition at the phylum level. **(B)** Microbial composition at the genus level. All data were normally distributed and analyzed by one-way ANOVA. Con: control group, Mod: model group, L-MRW: low dose of MRW group, M-MRW: medium dose of MRW group, and H-MRW: high dose of MRW group.

The structure and composition of colon microflora are shown in Figure 3A,B: At the phylum level, the top two phyla among all treatments are *Firmicutes* and *Bacteroidota*. Compared to the control rats, the relative abundance of *Firmicutes* was lowered and the relative abundance of *Bacteroidota* was increased in the STC rats. After the treatment with MRW, the relative abundance of *Firmicutes* was increased and the relative abundance of *Bacteroidota* was decreased in the L-MRW, M-MRW, and H-MRW rats, compared with the STC rats. At the genus level, compared to the control rats, four taxa significantly decreased in relative abundance and two taxa significantly increased in relative abundance. In these taxa, *Lactobacillus* was an increased taxon and *Prevotella* was a decreased taxon in the L-MRW, M-MRW, and H-MRW rats, compared with the STC rats. *Lactobacillus* belongs to Firmicutes and *Prevotella* belongs to Bacteroidota, and the most abundant significantly altered genus taxa were also *Lactobacillus* and *Prevotella*, indicating that the phylum level change might be mostly contributed by *Lactobacillus* and *Prevotella*. Therefore, the increase in *Lactobacillus* and the decrease in *Prevotella* were related to improvement of constipation in the STC rats with MRW treatments.

**FIGURE 4:**
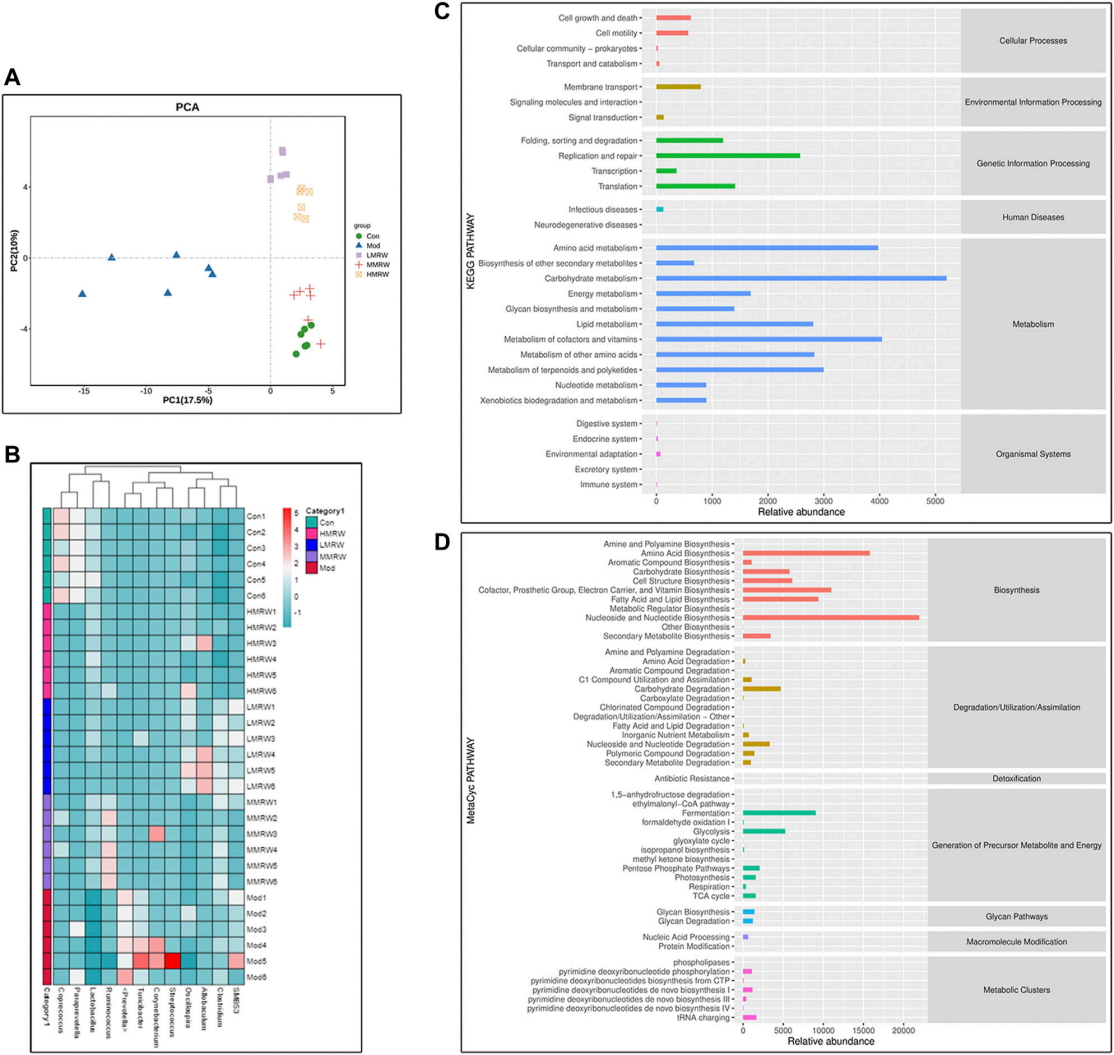
Similarity analysis of the colonic microbial structure and potential microbial pathway analysis of colonic microflora. **(A)** PCA. **(B)** Heatmap of genera level community composition. **(C)** KEGG results. **(D)** MetaCyc results. Con: control group, Mod: model group, L-MRW: low dose of MRW group, M-MRW: medium dose of MRW group, and H-MRW: high dose of MRW group.

Results from the principal component analysis (PCA) are shown in Figure 4A; under the principal component PC1 condition (17.5%), L-MRW, M-MRW, and H-MRW groups were close to the control group and the model group was far from the control group, suggesting that the colon microflora structure of the L-MRW, M-MRW, and H-MRW groups were more similar to the control group; in them, the L-MRW group was the closest to the control group. A heatmap of genera level community composition combined with a cluster analysis is shown in Figure 4B; it also shows that the colonic microbial structure of the L-MRW, M-MRW, and H-MRW groups are more similar to the control group. KEGG and MetaCyc analyses’ results used for exploring potential microbial pathways are shown in Figure 4C,D; the intestinal microflora was mainly enriched in the metabolic (analyzed by KEGG) and biosynthesis functions (analyzed by MetaCyc). The main metabolism pathways (Relative abundance ≥2500) analyzed by the KEGG were amino acid metabolism, carbohydrate metabolism, lipid metabolism, metabolism of cofactors and vitamins, metabolism of other amino acids, and metabolism of terpenoids and polyketides, and the main biosynthesis pathways (Relative abundance ≥7,500) analyzed by the MetaCyc were amino acid biosynthesis; cofactor, prosthetic group, electron carrier, and vitamin biosynthesis; fatty acid and lipid biosynthesis; and nucleoside and nucleotide biosynthesis. Therefore, the metabolism and biosynthesis of amino acid and lipid might play an important role in regulating constipation with MRW treatments.

### Effects of MRW on Short-Chain Fatty Acids (SCFAs) in Colonic Contents of STC Rats, and the Spearman Correlation Analysis Between the SCFAs and the Colonic Microflora

**FIGURE 5:**
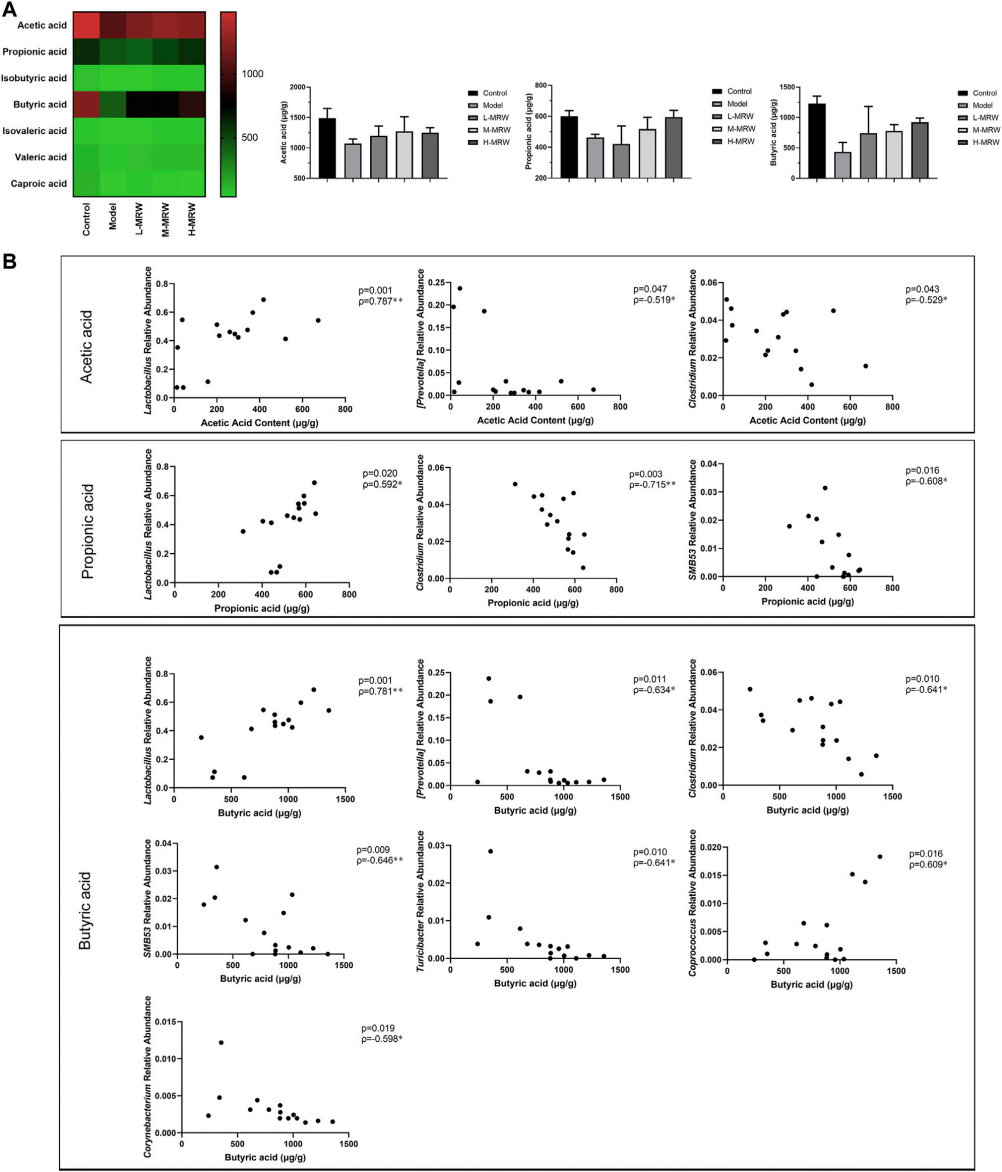
Effects of MRW on short-chain fatty acids (SCFAs) in colonic contents of the STC rats and Pearson correlations. **(A)** SCFAs’ results. **(B)** Pearson correlations. The data were normally distributed and analyzed by the Pearson correlation analysis.

In this study, we detected seven SCFAs (acetic acid, propionic acid, isobutyric acid, butyric acid, isovaleric acid, valeric acid, and caproic acid) in colon contents. As shown in Figure 5A, the contents of acetic acid, propionic acid, and butyric acid most obviously changed among the seven SCFAs. Compared to the control rats, the contents of acetic acid, propionic acid, and butyric acid were reduced in the STC rats (*p* > 0.05). After the treatment with MRW, the contents of acetic acid, propionic acid, and butyric acid were increased (*p* > 0.05). Finally, we performed a Pearson’s correlation analysis between the SCFAs (acetic acid, propionic acid, and butyric acid) and the top 12 dominant microflora at the genus level. As shown in Figure 5B, the relative abundance of *Lactobacillus* was positively correlated with either content among acetic acid, propionic acid, and butyric acid, and the relative abundance of *Clostridium* was negatively correlated. Moreover, the content of acetic acid was negatively correlated with the relative abundance of *Prevotella*, the content of propionic acid was negatively correlated with the relative abundances of *SMB53*, and the content of butyric acid was negatively correlated with the relative abundances of *Prevotella*, *SMB53*, *Turicibacter*, and *Corynebacterium* and positively correlated with the relative abundances of *Coprococcus*.

## Discussion

STC has become a common and frequently occurring disease in today’s society and is characterized by the slow colon peristalsis and the delayed expulsion of intestinal contents (Tian et al., 2017). STC affects normal life and even causes other intestinal diseases (Zhang et al., 2018). In the present study, to elucidate the mechanism of the laxative effect of MRW, we here explored the regulation of MRW on the 5-HT pathway, intestinal microflora, and intestinal metabolism in the colon of STC rats. The main findings of our study are as follows: 1) MRW improved the constipation of the STC rats *via* increasing intestinal fluid accumulation and intestinal motility; 2) MRW improved the colonic mucus barrier in the STC rats *via* increasing the goblet cells number, Muc-2 protein expression, and colonic mucus layer thickness; 3) MRW activated the 5-HT pathway; 4) MRW improved colonic microflora of the STC rats mainly by increasing *Lactobacillus* and decreasing *Prevotella*; 5) MRW regulated SCFAs’ metabolism in the STC rats, mainly by increasing the contents of acetic acid, propionic acid, and butyric acid, and the relative abundance of *Lactobacillus* was positively correlated with either content among acetic acid, propionic acid, and butyric acid.

The pathogenesis of STC involves various mechanisms. The loperamide-induced rat model of constipation was characterized by decreased fecal pellets, fecal water content, gastrointestinal transit ratio, and fecal SCFA levels, accompanied by an imbalance in intestinal microflora (Liu and Zhi, 2021). Thus, the study established the loperamide-induced rat model to explore the possible mechanisms of MRW in improving constipation. Our data revealed that MRW increased the body weight and the number, weight, and water content of fecal pellets in the STC rats and significantly alleviated constipation, which is consistent with the results of the previous study (Zhan et al., 2020). We also found the data of 24-h fecal number, 24-h wet fecal weight, 24-h dry fecal weight, fecal water content, and intestinal propulsion fitted the linear model, suggesting that MRW might improve the STC in a dose-dependent manner. Moreover, H-MRW and positive groups have shown almost the same effect.

The intestinal mucosal barrier integrity is necessary to maintain normal intestinal functions (Huang et al., 2020). The thick mucus covering the epithelium is the primary defensive layer separating microbiota from epithelial cells, which is synthesized by goblet cells (Johansson and Hansson, 2016). Gel-forming mucin2 (Muc-2) is the major building block of mucus, which accounts for up to 80% of the mass of mucin molecules, and Muc-2 is the reason for the gel-like properties of mucus (Johansson et al., 2008; Fu et al., 2011). Moreover, the intestinal gel-forming mucins are mainly from goblet cells (Huang et al., 2020). In our study, a fewer goblet cells number, thinner AB/PAS mucus layer, and lower Muc-2 protein expression in the colonic tissue of STC rats were observed. With MRW treatment, there was an increase in the goblet cells number, mucus layer, and Muc-2 protein expression in three MRW-treated groups, and H-MRW had the best improvement effect on those in three doses MRW. Our data showed that MRW improved the colonic mucus barrier in the STC rats *via* increasing the goblet cells number, Muc-2 protein expression, and colonic mucus layer thickness.

The disorder of the intestinal microflora is one of the characteristics of constipation patients (Tian et al., 2020). The intestinal microflora of chronic functional constipation (CFC) patients is abnormal in terms of numbers and composition (Huang et al., 2018). Recent studies show that there is a downregulation in the relative abundance of *Bifidobacterium* and *Lactobacillus* and an upregulation in the relative abundance of *Bacteroidetes*, *Fusobacterium*, and *Enterobacter* in CFC patients (Khalif et al., 2005; Pimentel and Lembo, 2020). Therefore, it is meaningful to explore whether the STC can be improved by MRW treatment by regulating intestinal microflora. Our data showed that the relative abundance of Firmicutes was increased and the relative abundance of Bacteroidota was decreased in the L-MRW, M-MRW, and H-MRW rats, compared with the STC rats at the phylum level, and the relative abundance of *Lactobacillus* was increased and the relative abundance of *Prevotella* was decreased in the L-MRW, M-MRW, and H-MRW rats, compared with the STC rats at the genus level. These indicated that MRW affected the composition and quantity of intestinal microbes by increasing the proliferation of beneficial bacteria and inhibiting the proliferation of harmful bacteria in the gut. In addition, the results of KEGG and MetaCyc analyses showed that the metabolism and biosynthesis of amino acid and lipid might play an important role in regulating constipation with MRW treatments. So, there is an interesting problem: “Whether there are some amino acids and lipids in MRW which can regulate the intestinal function directly?” However, we did not found related researches. Therefore, exploring amino acids and lipids contents of MRW and its role in MRW treatment for STC may be the next step in our study. One ability of the intestinal bacteria is to produce SCFAs, and the process is affected by the number of bacteria, pH, and substrate in the intestine (Liu and Zhi, 2021). SCFAs play an important role in the physiological metabolic processes *in vivo*. Acetic acid acts on upregulating the barrier function of host intestinal epithelial cells (Fukuda et al., 2011; Jiang et al., 2020); propionate acts on reducing fat production, serum cholesterol levels, and carcinogenic effects in other tissues (Hosseini et al., 2011; Jiang et al., 2020); and butyrate is a major source of metabolic energy in the large intestine and helps in maintaining the integrity of the large intestine, control intestinal inflammation, and support genomic stability (Jiang et al., 2020). Our study showed that MRW promoted the secretion of SCFAs in STC rats and increased the contents of acetic acid, propionic acid, and butyric acid to relieve constipation. Moreover, we found that the relative abundance of *Lactobacillus* was positively correlated with either content among acetic acid, propionic acid, and butyric acid, and the relative abundance of *Clostridium* was negatively correlated. These indicated that the improvement of the contents of acetic acid, propionic acid, and butyric acid to relieve constipation was connected to the relative abundance of *Lactobacillus* and *Clostridium* under the MRW treatment.

SCFAs not only stimulate water and electrolyte absorption and potentiate the proliferation of epithelial cells but also promote ileal propulsive contractions by causing prolonged propagated contractions and discrete clustered contractions (Wang G. et al., 2017; Wang L. et al., 2017; Lan et al., 2020). The mechanisms for SCFAs promoting gut motility may be related to the 5-HT pathway (Spiller, 2018; Russick et al., 2020). In the gastrointestinal tract, 5-HT contributes to electrolyte secretion and absorption, blood flow, perception of nausea or pain, and intestinal motility (Manzella et al., 2018). Recently, it was shown that the secretion of 5-HT in the gut is regulated by the intestinal microflora, and the SCFAs produced by the intestinal microflora could promote 5-HT levels in enterochromaffin cells in the epithelia (Yano et al., 2015; El Aidy et al., 2017). Tryptophan hydroxylase (TPH) is a rate-limiting enzyme for 5-HT synthesis and controlled the biosynthesis rate of 5-HT (Kim et al., 2018; Abad et al., 2020). Moreover, when 5-HT is uptaken in the cell, 5-HT is degraded into 5-hydroxyindoleacetic acid (5-HIAA) by monoamine oxidases (Manzella et al., 2018). 5-HT_4_R is a constitutively active Gs-coupled 5-HT receptor that could activate the production of cyclic adenosine monophosphate (cAMP) and the protein kinase A (PKA) pathway even in the absence of an agonist (Liu et al., 2019). The cystic fibrosis transmembrane conductance regulator (CFTR) is a cAMP-regulated anion channel required for adequate NaCl/fluid and bicarbonate secretion. Lack of CFTR function will lead to an acidic poorly hydrated intestinal environment (De Lisle and Borowitz, 2013), and it is considered a reason for causing the accumulation of mucus in the intestine, leading to bacteria colonization and overgrown, and leading to an inflammatory response (Norkina et al., 2004; De Lisle and Borowitz, 2013). In our study, we observed that the MRW increased the levels of 5-HT, 5-HIAA, 5-HT_4_R, CFTR, cAMP, and PKA in the colon tissue of the STC rats, indicating that MRW might activate the 5-HT pathway to promote the intestinal peristalsis and improve adequate NaCl/fluid and bicarbonate secretion.

## Conclusion

Our study further showed that MRW could improve constipation in STC rats *via* regulating the intestinal microflora structure and improving SCFAs’ secretion. The relative abundance of *Lactobacillus* and *Clostridium* were the main regulating microflora of MRW. The present study provided new evidence for the therapeutic effect of MRW from the intestinal microflora perspective.

## Data Availability

The datasets presented in this study can be found in online repositories. The names of the repository/repositories and accession number(s) can be found below: https://www.ncbi.nlm.nih.gov/bioproject/PRJNA792032.
