# Supplementary material for: Effects of Maren Pills on the Intestinal Microflora and Short-Chain Fatty Acid Profile in Drug-Induced Slow Transit Constipation Model Rats
Source: Front Pharmacol. 2022 Apr 12;13:804723. doi: 10.3389/fphar.2022.804723 (PMC9039019; doi:10.3389/fphar.2022.804723)
Supplement: Supplementary file 1 [file Table1.docx]

**Table S1 The chemical compositions (≥1%) results of MRW detected by the UPLC-MS**

| Title | RT (min) | Area | Adduct | Reference m/z | Formula | Ontology | The proportion of content (%) |
| --- | --- | --- | --- | --- | --- | --- | --- |
| C12-AS (TENTATIVE) | 10.432 | 1,897,327.50 | [M-H]- | 265.15 | C12H26O4S | Sulfuric acid monoesters | 11.32% |
| Dodecyl sulfate | 9.901 | 1,319,967.75 | [M-H]- | 265.15 | C12H26O4S | Sulfuric acid monoesters | 7.88% |
| 4-PYRIDOXATE | 10.834 | 1,302,801.00 | [M+H]+ | 184.06 | C8H9NO4 | Pyridinecarboxylic acids | 7.77% |
| S(8-8)S hexoside | 6.040 | 570,834.63 | [M-H]- | 579.21 | C28H36O13 | Lignols | 3.41% |
| Methyl 3-(3-methylbut-2-enyl)-1,4-bis[[3,4,5-trihydroxy-6-(hydroxymethyl)oxan-2-yl]oxy]naphthalene-2-carboxylate | 6.290 | 538,905.38 | [M-H]- | 609.22 | C29H38O14 | Phenolic glycosides | 3.22% |
| Diallyl Sulfide | 6.275 | 477,900.97 | [M+H]+ | 153.01 | C6H10S | Allyl sulfur compounds | 2.85% |
| Methyl 2-(4a,6,10a-trihydroxy-1-methyl-5,10-dioxo-3,4-dihydro-1H-benzo[g]isochromen-3-yl)acetate | 9.123 | 448,058.41 | [M+H]+ | 389.06 | C17H18O8 | Naphthopyranones | 2.67% |
| 9-stearolic acid | 13.631 | 410,644.06 | [M-H]- | 279.26 | C18H32O2 | Long-chain fatty acids | 2.45% |
| 2',5'-Dihydroxy-4-Methoxychalcone | 9.782 | 405,396.66 | [M-H]- | 269.08 | C16H14O4 |  | 2.42% |
| 5Alpha-Cholestan-3Beta-Ol-6-One | 14.146 | 383,161.47 | [M-H]- | 401.28 | C27H46O2 |  | 2.29% |
| Fallacinol | 6.153 | 344,127.28 | [M+H]+ | 301.05 | C16H12O6 | Anthraquinones | 2.05% |
| (4E)-8-hydroxy-4-(1-hydroxypropan-2-ylidene)-10-oxatricyclo[7.2.1.0?,?]dodecane-8-carboxylic acid | 10.352 | 320,808.75 | [M-H]- | 281.14 | C15H22O5 | Alpha hydroxy acids and derivatives | 1.91% |
| 3-(3,4-dihydro-2H-benzo[b][1,4]dioxepin-7-yl)-2,8-dimethyl-4-oxo-4H-chromen-7-yl acetate | 8.707 | 316,153.44 | [M+H]+ | 403.11 | C22H20O6 | Isoflavones | 1.89% |
| Isoflavone base + 2O, O-MalonylHex | 5.099 | 306,302.59 | [M+H]+ | 503.12 | C24H22O12 | Isoflavone O-glycosides | 1.83% |
| Tunicamyin C putative | 9.003 | 275,315.94 | [M+H]+ | 817.41 | C37H60N4O16 |  | 1.64% |
| Pachyrrhizin | 9.469 | 258,387.64 | [M+H]+ | 359.05 | C19H12O6 | Isoflav-3-enones | 1.54% |
| Rutin | 6.234 | 253,665.19 | [M+Na]+ | 633.14 | C27H30O16 |  | 1.51% |
| 5-methoxy-3-methyl-4-[(2S,3R,4S,5S,6R)-3,4,5-trihydroxy-6-[[(2R,3R,4S,5S,6R)-3,4,5-trihydroxy-6-(hydroxymethyl)oxan-2-yl]oxymethyl]oxan-2-yl]oxy-3H-benzo[f][2]benzofuran-1-one | 7.409 | 240,963.44 | [M+H]+ | 607.14 | C26H32O14 | Phenolic glycosides | 1.44% |
| Phillyrin | 5.396 | 231,660.22 | [M-H]- | 579.21 | C27H34O11 | Lignols | 1.38% |
| 1-(2,4-dihydroxyphenyl)-2-(3,5-dimethoxyphenyl)propan-1-one | 7.625 | 212,676.80 | [M-H]- | 301.11 | C17H18O5 | Alpha-methyldeoxybenzoin flavonoids | 1.27% |
| C9H8O2 | 6.592 | 211,538.64 | [M+H]+ | 131.05 | C9H8O2 | Formula predicted | 1.26% |
| 2-(3,4-dihydroxyphenyl)ethyl (1S,4aR,7aR)-4a-hydroxy-7-methyl-5-oxo-1-[(2S,3R,4S,5S,6R)-3,4,5-trihydroxy-6-(hydroxymethyl)oxan-2-yl]oxy-1,6,7,7a-tetrahydrocyclopenta[c]pyran-4-carboxylate | 4.900 | 208,807.02 | [M-H]- | 525.20 | C24H30O13 | Iridoid O-glycosides | 1.25% |
| (2S,3R,4S,5R,6R)-2-(4-chlorophenoxy)-6-(hydroxymethyl)tetrahydro-2H-pyran-3,4,5-triol | 8.339 | 199,894.45 | [M+H]+ | 313.05 | C12H15ClO6 | Phenolic glycosides | 1.19% |
| Ascorbic acid | 6.275 | 181,721.75 | [M+H]+ | 177.04 | C6H8O6 | Butenolides | 1.08% |
