# Supplementary material for: Effects of Maren Pills on the Intestinal Microflora and Short-Chain Fatty Acid Profile in Drug-Induced Slow Transit Constipation Model Rats
Source: Front Pharmacol. 2022 Apr 12;13:804723. doi: 10.3389/fphar.2022.804723 (PMC9039019; doi:10.3389/fphar.2022.804723)
Supplement: Supplementary file 2 [file DataSheet1.docx]

**Supplementary material:**

**UPLC-MS detection steps:**

1. The extraction of MRW active components:

After MRW grinding, 0.10g sample was added with 1mL methanol, followed by ultrasound for 30min and centrifugation for 10 min (4℃, 12000rpm). then 10uL of supernatant was taken for sample detection.

1. Instruments: The Shimadzu ULTRA High Performance Liquid chromatograph (LC-30) is connected to the SCIEX 5600+ mass spectrometer.
2. Analysis conditions of UPLC-MS:

①The chromatographic column:

Waters BEH C18 (150×2.1 mm, 2.5 µm). The column temperature was 40℃. The flow rate was 0.3 [ml/min].

②The mobile phase:

1. Equate="0.1% HCOOH-H_2_O "
2. Equate="acetonitrile"

③The chromatographic conditions (Time:min, Parameter):

0 min: A: 95%, B: 5%

2 min: A: 95%; B: 5%

10 min: A: 5%; B: 95%

13 min: A: 5%; B: 95%

15 min: A: 95%; B: 5%

④The mass spectrometry conditions:

The positive and negative ionization modes of electrospray ionization (ESI) were used for detection. ESI Source conditions are as follows:

Ⅰ. Ion Source Gas1(Gas 1): 50;

Ion Source Gas2(Gas 2): 50;

Curtain Gas (CUR): 25;

Ⅱ. Source Tempreture: 500℃ (positive ionization) and 450℃ (negative ionization);

Ⅲ. Ion Sapary Voltage Floating (ISVF):5500V (positive ionization) and 4400V (negative ionization);

Ⅳ. TOF MS Scan range: 100-120 Da;

Ⅴ. Product ion scan range: 50-1200 Da;

Ⅵ. TOF MS Scan accumulation time: 0.2s;

Ⅶ. Product ION scan accumulation time: 0.1s;

Ⅷ. The second order mass spectrometry is obtained by Information Dependent Acquisition (IDA) and has a high-sensitivity mode: Declustering potential(DP): ±60V; Collision Energy: 35±15eV.
